# Non-rapid eye movement sleep and isoflurane-induced anesthesia show divergent subcortical connectivity patterns during transition phases in mice

**DOI:** 10.1093/sleep/zsaf287

**Published:** 2025-09-24

**Authors:** Leesa Joyce, Rachel Nuttall, Matthias Kreuzer, Gerhard Rammes, Gerhard Schneider, Thomas Fenzl

**Affiliations:** Department of Anesthesiology & Intensive Care, School of Medicine and Health, Technical University of Munich, Munich, Germany; Department of Anesthesiology & Intensive Care, School of Medicine and Health, Technical University of Munich, Munich, Germany; Department of Anesthesiology & Intensive Care, School of Medicine and Health, Technical University of Munich, Munich, Germany; Department of Anesthesiology & Intensive Care, School of Medicine and Health, Technical University of Munich, Munich, Germany; Department of Anesthesiology & Intensive Care, School of Medicine and Health, Technical University of Munich, Munich, Germany; Department of Anesthesiology & Intensive Care, School of Medicine and Health, Technical University of Munich, Munich, Germany

**Keywords:** general anesthesia, LFP, EEG, sleep/wake promoting pathway, VLPO, LC, functional connectivity, coherence, inter site phase clustering, Granger causality

## Abstract

**Study Objectives:**

Neural dynamics underlying anesthesia-induced unconsciousness are not fully understood. Given the parallels between natural sleep and anesthesia-induced unconsciousness, it becomes imperative to understand the neuronal mechanisms behind these two distinct yet seemingly interconnected states. This study investigated the interplay between sleep/wake-promoting nuclei during WAKE/non-rapid eye movement sleep (NREMS) transitions and the reversible loss and recovery of responsiveness (LOR/ROR) induced by isoflurane.

**Methods:**

Local field potentials (LFPs) were recorded from the ventrolateral preoptic area (VLPO) and locus coeruleus (LC) in mice alongside chronic electroencephalogram-recordings. After baseline recordings, a slow induction regime of isoflurane anesthesia followed. Functional connectivity between VLPO and LC during NREMS, WAKE, LOR, and ROR was studied using coherence, inter-site phase clustering, and Granger causality analyses.

**Results:**

LFP data revealed an increase in coherence between VLPO and LC during NREMS and a decrease during WAKE. Coherence decreased after LOR. During ROR, coherence did not change. Phase clustering between VLPO and LC increased during NREMS and decreased during WAKE, while across LOR/ROR transitions did not vary. Granger between VLPO and LC during WAKE/NREM transitions demonstrated bidirectional influences of the two nuclei. VLPO Granger caused LC during NREMS. In the slow-wave frequency, the Granger index from LC to VLPO decreased during NREMS, while after ROR, the Granger index from VLPO to LC increased.

**Conclusions:**

The present study revealed functional connectivity patterns between VLPO and LC during sleep and isoflurane anesthesia, suggesting that these processes partly do not share similar functional connectivity patterns for the two nuclei.

## Graphical Abstract

**Graphical Abstract:**
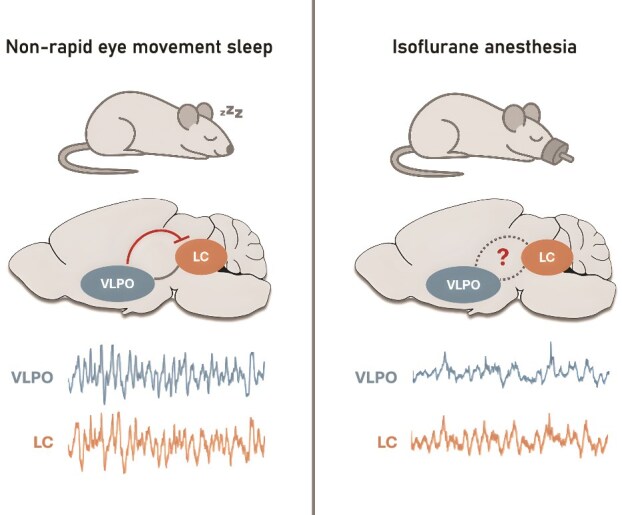

## Teaser

**Statement of Significance** Non-rapid eye movement sleep (NREMS) like electroencephalogram patters during loss of responsiveness (LOR) and especially during recovery of responsiveness (ROR) after general anesthesia seem to be protective against an emergence delirium and also shorten the time spent in the post-anesthesia care unit. Understanding the potential systemic contribution of the sleep/wake promoting pathway during LOR/ROR may help to control for natural, sleep-like ROR. The present study investigated functional connectivity between two major nuclei of the sleep/wake promoting pathway, namely the locus coeruleus (LC) and the ventrolateral preoptic area (VLPO) during the natural transition from WAKE to NREMS and during LOR/ROR. The findings suggest functional activity patterns between LC and VLPO are at least partly different in sleep transitions versus LOR/ROR.

The mechanisms governing altered states of consciousness, such as natural sleep and general anesthesia (GA), are of paramount interest due to their respective roles in restorative processes [1–3] and medical practices [4]. Sleep, a naturally recurring behavior and GA, a pharmacologically induced state represent partly different physiological states, induced and maintained by at least partly different electrophysiological and molecular mechanisms [5, 6]. But both states also share some intriguing similarities in their neural mechanisms and behavioral manifestations [7–9].

While all anesthetics have similar behavioral endpoints [10], most of them exhibit unique properties and mechanisms of action [11]. Extensive evidence from studies indicates that GA affects specific networks in the brain rather than causing overall suppression [12]. Because of the parallels between sleep and GA-states, starting from Alexander Fleming in 1855 [13], researchers have explored the idea that different GA- inducing drugs might induce hypnotic effects by targeting neural circuits in the endogenous sleep/wake promoting pathways. Some studies have shown that GA, particularly those targeting gamma-aminobutyric acid (GABA) pathways such as isoflurane, sevoflurane and propofol influence sleep-promoting pathways [11, 14, 15], eventually giving rise to the shared circuit hypothesis. *N*-methyl-*D*-aspartate (NMDA) receptor antagonists like ketamine, xenon, and nitrous oxide may engage these pathways differently, by altering excitatory neurotransmission rather than directly enhancing GABAergic inhibition [16–18].

However, the shared circuit hypothesis has been questioned in recent years suggesting that sleep and GA might only share partial similarities [7, 11], with reports of endogenous sleep circuitry being engaged only for sedation through GABA(A) receptors as well as NMDA receptor antagonists and not for GA [15, 19]. Some studies proposed that GA directly influences the neural substrates responsible for consciousness, and the endogenous sleep circuitry is neither adequate nor essential for the induction of GA [6, 20–22].

Among the known sleep/wake regulating nuclei, the ventrolateral preoptic area (VLPO) suppresses regional activity that drives arousal and plays a crucial role in promoting sleep [23]. Galanin and GABAergic neurons from the VLPO project to arousal regions like the histaminergic tuberomammillary nucleus (TMN), dorsal raphe (DR), and locus coeruleus (LC) [24]. During non-rapid eye movement sleep (NREMS), the VLPO inhibits these arousal nodes, effectively silencing them, while in the waking state, the LC, TMN, and DR strongly inhibit the VLPO through synaptic release of noradrenaline, histamine and serotonin [25]. Due to the crucial roles and the state-dependent (NREMS and WAKE) reciprocal inhibition, VLPO and LC are prominent candidates generally representing the sleep/wake circuitry.

Isoflurane is known to enhance the activity of inhibitory receptors such as postsynaptic GABA_A_ receptors [26], specifically also at the level of VLPO [15]. The hypnotic effect of isoflurane has been known to be modulated by LC as well. Activation of LC is known to delay isoflurane induction [27] and promote isoflurane emergence [28]. Furthermore, several studies have pointed out similarities between slow wave activity (SWA) observed during NREMS and the SWA induced by isoflurane [29]. It remains unclear whether these area-specific corresponding processes are simply coincidental or are a result of an unequivocal physiological brain state sharing VLPO and LC activity.

The precise role of the sleep/wake promoting pathway in drug-induced unconsciousness (or rather unresponsiveness for animal research, as outlined in the discussion section) during GA remains unclear. Therefore the present study investigated neuronal functional connectivity patterns between the VLPO and LC in mice and examined how such patterns change during natural transitions between WAKE and NREMS and during isoflurane anesthesia-induced loss of responsiveness (LOR) and recovery of responsiveness (ROR).

## Materials and Methods

### Animals and housing

Twelve male C57BL/6N mice (Charles River Laboratories GmbH, Germany) aged between 12 and 18 weeks (BW: 24–27 g) were used for the experiments. The animals were housed individually under a 12/12-h light/dark cycle (lights ON/OFF: 09:00 am/09:00 pm, temperature: 22°C ± 2°C, humidity: 55% ± 10%) with *ad libitum* access to food and water. To minimize phase variability and ensure circadian synchronization, mice were relocated from the animal facility to the laboratory 3 days before surgery, during the maintenance hour (08:00–09:00 am, lights—ON, active phase). All mice were kept under similar living conditions. On the third day, mice received analgesia (carprofen) via drinking water and surgery was performed on the fourth day. All experimental procedures were approved by the Committee of Animal Health Care of the State of Upper Bavaria, Germany (ROB-55.2–2532.Vet_02–19–121). Care of laboratory animals and experiments were performed in accordance with the recommendations of the European Union for the care and use of laboratory animals and in accordance with ARRIVE guidelines [30]. The study was not pre-registered with the Open Science Framework.

### Surgery and electrode implantation

For the electrode implantations, anesthesia induction was performed in an acrylic glass chamber with 4 Vol.-% isoflurane (CP-Pharma Handelsgesellschaft GmbH, Germany). To assess LOR, the mouse was carefully placed on its dorsal side by gradually inclining the chamber. After confirming LOR within the chamber, the mouse was transferred to a stereotaxic frame (Leica Mikrosysteme Vertrieb GmbH, Germany). Throughout the surgery, anesthesia was maintained at 1.6–2.0 Vol.-% isoflurane in room air at a flow rate of 192 mL/min. Body temperature of 37°C was maintained with a homeothermic monitoring system (Harvard Apparatus, USA). For analgesia, Carprofen (4 mg/kg body weight; Zoetis, Germany) was administered subcutaneously prior to surgery, while Lidocaine Hydrochloride (2 per cent; bela-pharm GmbH & Co. KG, Germany) was applied locally to the incision sites during the procedure. Following shaving of the head, incisions were made to expose the upper cranium, and the periosteum was removed. Three epidural electroencephalogram (EEG) electrodes were implanted at specific coordinates (left frontal: AP +1.78 mm, ML −1.17 mm; left parietal: AP −2.70 mm, ML −1.57 mm; additional ground electrode: AP +0.62 mm, ML −3.02 mm), alongside an EMG electrode placed on the nuchal muscle. Additionally, local field potential (LFP) electrodes were implanted, targeting the VLPO (AP +0.01 mm, ML −0.65 mm, DV −5.75 mm) and the LC (AP −5.41 mm, ML −0.82 mm, DV −3.78 mm). A printed circuit board socket (Preci-Dip, series 861, Delémont, Switzerland) holding the electrodes was permanently secured onto the cranium. The socket was attached with dental cement (Paladur, Kulzer GmbH, Germany) and two jeweler’s screws. The EEG and EMG electrodes were fabricated from 24 K gold wires (diameter: 150 μm, Haefner & Krullmann GmbH, Germany), while the LFP electrodes were made from stainless-steel wires with perfluoroalkoxy alkane insulation (bare wire diameter: 76 μm; coated wire diameter: 140 μm, Science Products GmbH, Germany). Postoperative analgesia (Carprofen, 0.067 mg/mL) was provided for three days via drinking water.

### Experimental design

After surgery, mice were allowed to recover for 10 days giving them 2 weeks for entrainment (synchronization to light–dark cycle starting from 3 days before surgery, the day of surgery and 10 days of recovery post-surgery). Following the recovery period, continuous baseline EEG, EMG, and LFP data were recorded for two consecutive days starting at ZT 09:00 am. On each recording day, data were acquired for 23 h (12 h light period and 11 h dark period) where the last hour of the dark period was used for animal care and technical maintenance. On the third day, experimental anesthesia was administered at 09:00 am. EEG and LFP data were continuously recorded during baseline, the experimental anesthesia and the following 2 days. Except for the experimental anesthesia, the mice were allowed to behave freely in the home cage. At termination of the experiment, the mice were unplugged from the recording setup, and under isoflurane anesthesia, the positions of the LFP electrodes were marked through electrical lesions (4 μA for 5 min at each nucleus separately via the implanted LFP electrodes). Immediately after the lesions were made, the mice were perfused (4 percent PFA), and the brains were snap-frozen. The brains were later sliced and Nissl-stained to verify whether the LFP electrodes were on target (refer to Figures SS1–SS3) by localizing the electric lesions and matching them with a stereotaxic atlas [31].

### Experimental anesthesia

In the experimental setup, mice were anesthetized in a transparent acrylic chamber (volume: 0.006 m^3^) equipped with a hermetic seal, a heating pad, a system for monitoring body temperature (maintained at 37°C) and physiological oxygen saturation (O_2_ ≥ 92%; MouseOx Plus, Starr Life Science Corp., USA). The chamber featured a gas inlet, a gas vent, a gastight port for the EEG/LFP recording cable and a gas probe. Chamber airflow was maintained at 1.5 L/min with an inspiratory oxygen concentration of 0.5 [32] (CAPNOMAC Ultima, Datex Ohmeda, USA). Anesthesia was initiated simultaneously with data acquisition, starting at 0.1 per cent isoflurane, which was increased by 0.1 per cent every 2 min until a one-second burst suppression bout was observed in the live EEG. Subsequently, the isoflurane concentration was reduced by 0.1 per cent every 2 min until it reached 0 per cent. During the induction phase, the chamber was gently tilted every 2 min to assess LOR, with the time point recorded. The same method was used during the emergence phase to determine ROR.

### Data acquisition

Each mouse was connected to a tethered recording system comprising a headstage and recording cable with 1× amplification (custom-designed, npi electronics GmbH, Germany), integrated with a commutator (SL-20 model, Dragonfly R&D Inc., USA) mounted on a custom weight-neutral swivel system (Streicher M., Innsbruck, Austria) to enable unrestricted movement in the homecage. The EEG, EMG, and LFP signals were amplified independently by 1000× using a differential amplifier (DPA-2FL, npi electronics, Germany). EEG and EMG signals were band-pass filtered online between 0.1 and 100 Hz and sampled at 250 Hz, while LFP signals were band-pass filtered online between 0.1 and 1000 Hz and sampled at 2500 Hz (Power1401-3A board, Cambridge Electronic Design Ltd., UK) and Spike2 software (Cambridge Electronic Design Ltd., UK). Notch filters at 50 Hz were applied to all recording channels. A high-pass filter with a cutoff frequency of 0.5 Hz (Butterworth, first order) was used offline to eliminate low-frequency transients. No additional filtering was applied [33]. All data were subsequently imported into MATLAB (R2019a, MathWorks, USA) for preprocessing and analysis.

## Data Analyses

### Sleep data: sleep scoring and epoch selection

A LABVIEW-based (National Instruments, Austin, TX, USA), semi-automated sleep scoring software [34, 35] was applied to the EEG recordings. The signals from the left parietal EEG and the corresponding EMG were segmented into 4-second non-overlapping epochs. Each epoch was assigned to one of the three vigilance states: WAKE, NREMS and rapid eye movement sleep (REMS). The semi-automated sleep scores were manually reviewed by an experienced scorer to ensure accuracy of vigilance state assignments, especially at all vigilance state transitions. All epochs corresponding to transitions between WAKE and NREMS were labeled for further analyses.

To investigate the connectivity between VLPO and LC during transitions between WAKE and NREMS, 2-min signal segments were selected, centered around the transition point. Only transitions with at least 1 min of data from each state on either side of the transition were included in the analysis. Longer segments were not selected to minimize the impact of nonstationary dynamics and transient changes in sleep architecture, which may occur at a temporal resolution finer than that provided by conventional sleep scoring methods.

### Isoflurane data: epoch selection

Two-min segments were selected as the anesthesia protocol involved steps that are 2-min long with constant isoflurane dosage. To minimize artifacts associated with movement caused by experimental manipulations (box tilts), the 2-min intervals immediately before and after LOR and ROR were excluded. Consequently, a pre-LOR segment with an interval from −4 to −2 min relative to LOR, and a post-LOR segment as +2 to +4 min relative to LOR was defined. Similarly, pre-ROR and post-ROR segments were identified using the same criteria relative to the ROR time point.

### Functional connectivity analysis 1: coherence

The continuous LFP signals from VLPO and LC were extracted from the two-day baseline recordings and segmented into nonoverlapping 4-s epochs to ensure a sync in time with the corresponding EEG epochs and sleep scores. Coherence between the nuclei pair VLPO-LC was calculated using the inbuilt MATLAB function *mscohere*. Coherence was calculated within the 0.5–40 Hz range in steps of 0.5 Hz. The coherence values of epochs at each frequency for all selected transition segments were extracted. The coherence values were similarly computed for pre-LOR/post-LOR segments and pre-ROR/post-ROR segments.

### Functional connectivity analysis 2: inter site phase clustering

The time series were restructured into time-frequency domain by dividing the signal into bands from 0.5 to 40 Hz at a resolution of 0.5 Hz using a zero-phase finite-duration impulse response filter (FIR-filter, filter order: 5, roll-off rate: 30 dB/octave). The signals were then transformed into analytic signals using the Hilbert transform and then for each frequency band and time point, the instantaneous phase of the signal was calculated. The phase difference between the signals from two electrodes *x* and *y* was computed at each time point and frequency, and the inter site phase clustering (ISPC) was calculated using the formula:


$$ {ISPC}_{x,y}(f)={\left({e}^{i\ \left[\varphi \left(t,f,x\right)-\varphi \left(t,f,y\right)\right]}\right)}_t $$

Where Φ is the instantaneous phase, *t* represents time, *f* denotes frequency. For both the sleep data and isoflurane data, the ISPC between VLPO and LC was computed for each epoch at each frequency for all identified transition segments.

### Functional connectivity analysis 3: Granger causality

To calculate Granger causality (GC) (MATLAB-based Multivariate GC toolbox [33]) in the time-frequency domain, a vector autoregressive (VAR) model for the time series *X_t_* and *Y_t_* were computed for time *t*. Cross-spectral density matrix *S(f)* for frequency *f* was calculated where *S(f)* contained spectral components *S_XX_(f), S_YY_(f),* and *S_XY_(f)*. To determine the influence of *X_t_* on *Y*_*t*,_ the conditional power spectrum *S_Y|X_(f)* was computed (Equation 1).


(1) \begin{equation*} {S}_{Y\mid X}(f)={S}_{YY}(f)-\frac{{\left|{S}_{XY}(f)\right|}^2}{S_{XX}(f)} \end{equation*}

GC at frequency *f* was calculated (Equation 2).


(2) \begin{equation*} {F}_{X\to Y}(f)=\ln \left(\frac{S_{YY}(f)}{S_{Y\mid X}(f)}\right) \end{equation*}

Bivariate GC was calculated in the time-frequency domain for frequencies between 0.5 and 40 Hz with a 0.5 Hz resolution. For calculating GC using the auto-covariance-based method, the dataset was windowed into 4-s time series and each window was normalized by subtracting its mean and dividing by the *SD*. The stationarity was tested using the augmented Dickey-Fuller test [36]. The stationary time series were used to calculate the order of the VAR model using a methodology based on Akaike's Information Criterion (AIC) and the Bayesian Information Criterion (BIC) [37, 38]. Both AIC and BIC suggested model orders in the range between 18 and 22. For the model order to be high enough to provide a reliable model and low enough to allow for sufficient computational tractability, 22 was chosen as the model order for the VAR model estimations to calculate GC. For the autoregressive model, the autoregressive coefficients were calculated following Morf’s modified Locally Weighted Regression method [39].

### Statistical analysis

All statistics were computed using custom scripts in MATLAB—R2019a (MathWorks, USA). Graphical representations were created using MATLAB as well. The percentage change was calculated at each epoch for each frequency relative to the baseline which was defined as the average value of the state preceding the transition. The statistical tests were performed on the percentage changes and all tests were two-tailed where significance was achieved at a 95% confidence interval (CI) (α = 0.05, Wilcoxon signed rank test). To correct for multiple comparisons, the results were considered significant only if they occurred in clusters and over 3 epochs (minimum duration for stable vigilance states; derived from our sleep-scoring routine), as reported previously [40, 41]. Effect sizes were calculated as Cohen's d (d) and CIs were calculated with 95% significance level for each epoch at every frequency bin for all comparisons. The d and CI of significant results for each graph were reported as mean ± *SD* (μ ± σ).

All connectivity metrics are represented as group medians in time-frequency domains in the figures. In Figures 3 to 8, the top rows show the group medians of the metrics and the bottom rows show the group medians of the percentage change of the metrics. A percentage increase is represented in gradients of red and a percentage decrease is represented in gradients of blue. The clusters of significant changes in connectivity metrics are enclosed in thick black lines. Connectivity trajectories over time were smoothed using a five-point moving average.

## Results

**Figure 1:**
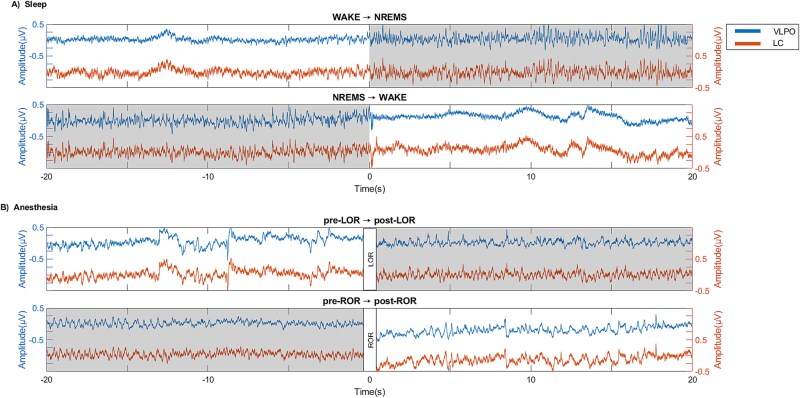
Randomly selected raw signals of VLPO (blue, left *y*-axis) and LC (orange, right *y*-axis) from transition blocks during (A) sleep and (B) anesthesia. Unresponsive states (NREMS, post-LOR, and pre-ROR are depicted with a grey background). The signals around LOR and ROR are discontinuous since the data was removed due to the movement artifacts caused by box tilts.

The transition blocks for sleep (WAKE → NREMS and NREMS → WAKE) and isoflurane anesthesia (pre-LOR → post-LOR and pre-ROR → post-ROR) transitions were chosen for coherence, ISPC and GC analyses. Figure 1 shows four examples of transition blocks.

### Sleep data analysis

The analysis was initiated by evaluating coherence between VLPO and LC activity across the entire 23-h of timeseries data for each baseline of every mouse, which included the recording from both the 12-h inactive (lights—ON) and the 11-h active (lights—OFF) phase. The results showed that VLPO–LC coherence consistently increased during NREMS irrespective of circadian phase. To assess specificity, coherence between another pair of nuclei (VPM–LC) was also examined as a control, for which no comparable state-dependent pattern was observed (refer to Figure SS4). These findings demonstrated that the increase in VLPO—LC coherence was reliably associated with the WAKE → NREMS transition and was not restricted to a particular circadian time.

**Figure 2:**
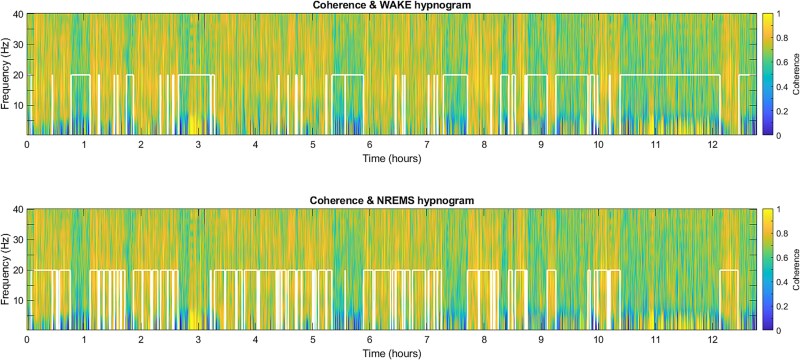
Exemplary graph for coherence between VLPO-LC electrode pairs from a representative mouse through ~12 h (*x*-axis) of baseline recording. Coherence was calculated for the frequencies 0.5–40 Hz (left *y*-axis). The color bar indicates the range of coherence from 0 to 1. The coherence plot is overlaid with a continuous trendline graph in white color for the WAKE hypnogram (top graph, WAKE at white line “high”) and NREMS hypnogram (bottom graph, NREMS at white line “high”). Coherence between VLPO and LC electrodes increased at the transition from WAKE → NREMS and decreased at the transition from NREMS → WAKE.

Based on this visual pre-analysis, coherence between VLPO and LC electrodes strictly increased at each transition from WAKE → NREMS and decreased strictly at each transition from NREMS → WAKE. A representative analysis for an individual mouse is shown in Figure 2. The corresponding sleep scores were overlaid (white trendline graphs) to visualize the vigilance state-dependent coherence between the nuclei pairs.

**Figure 3:**
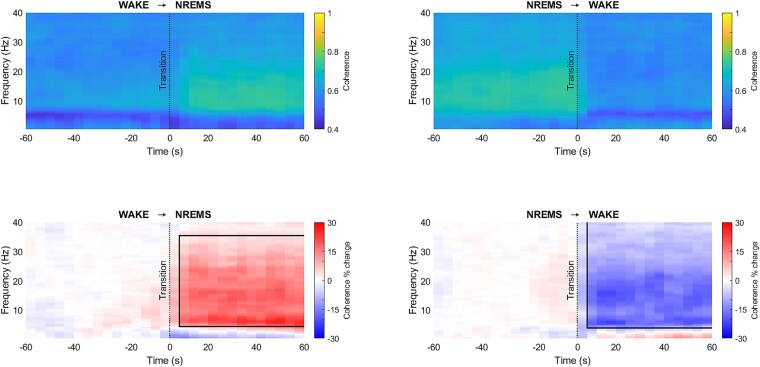
Coherence (color bar: Coherence, top graphs) between VLPO and LC electrodes for WAKE → NREMS (left) and NREMS → WAKE (right) transitions for frequencies between 0.5 and 40 Hz. Percentage change of coherence (color bar: Coherence per cent change, bottom graphs) across transitions was statistically compared (*n* = 12; significant changes are indicated with black bounding lines; red depicts an increase, and blue depicts a decrease).

The percentage change of coherence with respect to the reference was statistically compared for both WAKE → NREMS and NREMS → WAKE transitions for all the frequencies between 0.5 and 40 Hz (Figure 3).

A significant increase in the coherence percentage during NREMS around 5–35 Hz (Figure 3, bottom-left, d = 0.94 ± 0.26, 95% CI = [3.79% ± 14.04% to 35.85% ± 45.08%]) and a significant decrease in coherence percentage during WAKE around 4–40 Hz (Figure 3, bottom-right, d = −1.40% ± 0.38% to 95% CI = [−21.43% ± 7.29% to −7.85% ± 3.93%]) was identified. No significant difference in coherence in the slow wave frequency range was found.

**Figure 4:**
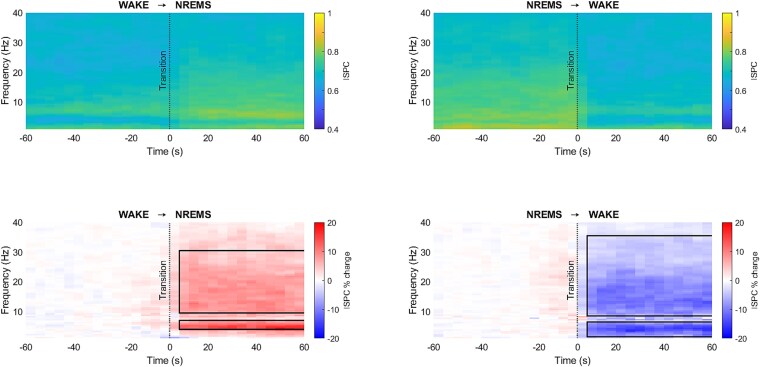
ISPC (colorbar: ISPC, top graphs) between VLPO and LC electrodes for WAKE → NREMS (left) and NREMS → WAKE (right) transitions for frequencies between 0.5 and 40 Hz. Percentage change of ISPC (colorbar: ISPC per cent change, bottom graphs) across transitions was statistically compared (*n* = 12; significant changes are indicated with black bounding lines; red depicts an increase, and blue depicts a decrease).

ISPC between VLPO and LC was computed for the selected transitions for each mouse and the percentage change of ISPC with respect to the reference was statistically compared for both WAKE → NREMS and NREMS → WAKE transitions for all the frequencies between 0.5 and 40 Hz (Figure 4). A significant increase in the ISPC percentage during NREMS around 3–7 and 10–30 Hz (Figure 4, bottom-left, d = 1.00 ± 0.20, 95% CI = [3.16 ± 1.79 to 15.31 ± 6.36]) was identified. During NREMS → WAKE transition, a significant decrease in ISPC percentage during WAKE was observed around 0.5–6 Hz and 9–35 Hz (Figure 4, bottom-right, d = −1.26 ± 0.40, 95% CI = [−13.10% ± 4.74% to −4.20% ± 2.46%]).

**Figure 5:**
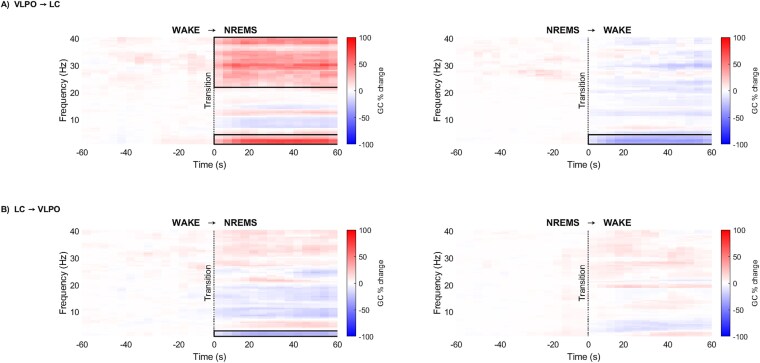
Bidirectional GC between VLPO and LC electrodes: (A) VLPO to LC and (B) LC to VLPO in the time-frequency domain during WAKE → NREMS transitions (left) and NREMS → WAKE transitions (right). Percentage change of GC (colorbar: GC per cent change) across transitions was statistically compared (*n* = 12; significant changes are indicated with black bounding lines; red depicts an increase, and blue depicts a decrease).

In WAKE → NREMS transitions, GC from VLPO to LC significantly increased during NREMS around 0.5–4 Hz and 21–40 Hz (Figure 5, top-left, d = 0.87 ± 0.21, 95% CI = [11.59% ± 10.67% to 78.81% ± 29.81%]). Conversely, in NREMS → WAKE transitions, the GC from VLPO to LC exhibited a reverse trend (compared to WAKE → NREMS transitions), with a significant decrease observed during WAKE only within the 0.5–4 Hz range (Figure 5, top-right, d = −2.11 ± 0.75, 95% CI = [−32.43% ± 9.38% to −16.46% ± 8.31%]). The GC from LC to VLPO during NREMS in WAKE → NREMS transitions significantly decreased around 0.5–3 Hz (Figure 5, bottom-left, d = −1.61 ± 0.50, 95% CI = [−31.59% ± 7.96% to −12.94% ± 5.89%]). The GC from LC to VLPO did not change significantly across NREMS → WAKE transitions (Figure 5, bottom-right).

### Isoflurane data analysis

**Figure 6:**
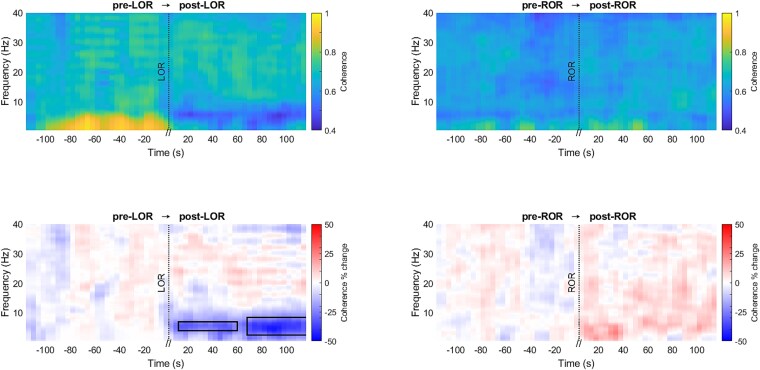
Coherence (colorbar: Coherence, top graphs) between VLPO and LC electrodes during LOR (left) and ROR (right) for frequencies between 0.5 and 40 Hz. Percentage change of coherence (colorbar: Coherence per cent change) across pre-LOR → post-LOR (bottom-left) and pre-ROR → post-ROR (bottom-right) transitions were statistically compared (*n* = 12; significant changes are indicated with black bounding lines, red depicts an increase, and blue depicts a decrease).

The coherence between VLPO and LC was computed across LOR for each mouse for the frequency range of 0.5–40 Hz (Figure 6, top-left). The coherence percentage change during pre-LOR was compared with post-LOR for all the twelve mice, clearly showing that coherence decreased during post-LOR, as compared to pre-LOR between 3 and 8 Hz (Figure 6, bottom-left, d = −0.97 ± 0.26, 95% CI = [−49.42% ± 8.27% to −9.84% ± 6.58%]).

The coherence between VLPO and LC across ROR was computed for each mouse for the frequency range of 0.5–40 Hz (Figure 6, top-right) and the coherence percentage change did not show significant differences between pre-ROR and post-ROR (Figure 6, bottom-right).

**Figure 7:**
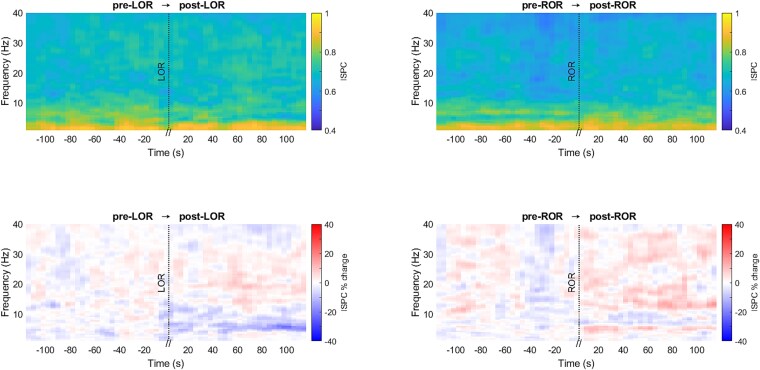
ISPC (colorbar: ISPC, top graphs) between VLPO and LC electrodes across LOR and ROR for frequencies between 0.5 and 40 Hz. Percentage change of ISPC (colorbar: ISPC per cent change) across pre-LOR → post-LOR (bottom-left) and pre-ROR → post-ROR (bottom-right) transitions was statistically compared (*n* = 12; significant changes are indicated with black bounding lines; red depicts an increase, and blue depicts a decrease).

ISPC between VLPO and LC was calculated during LOR and ROR (Figure 7). The ISPC percentages did not significantly change across transitions. ISPC values were notably high (above 0.8) for the slow wave frequency range.

**Figure 8:**
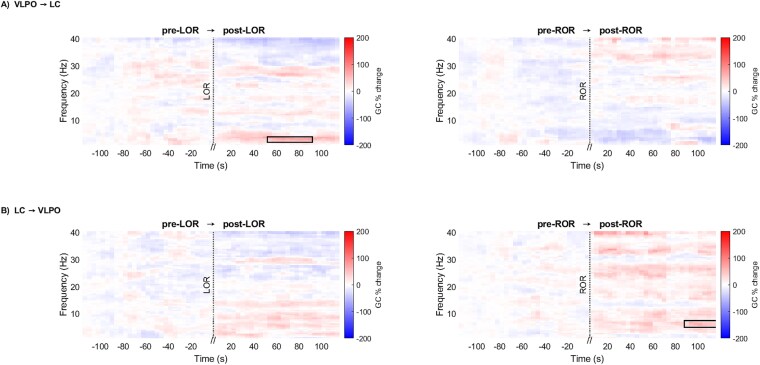
Bidirectional GC between VLPO and LC electrodes; (A) VLPO to LC and (B) LC to VLPO in time-frequency domain during LOR (left) and ROR (right). Percentage change of GC (colorbar: GC per cent change) across pre-LOR → post-LOR (left) and pre-ROR → post-ROR (right) transitions was statistically compared (*n* = 12; significant changes are indicated with black bounding lines; red depicts an increase, and blue depicts a decrease).

During LOR, the GC from VLPO to LC significantly increased in post-LOR around 1–4 Hz (Figure 8, top-left, bottom-left, d = 0.75 ± 0.04, 95% CI = [11.44% ± 4.16% to 129.28% ± 9.29%]). During ROR, the GC from VLPO to LC did not change significantly (Figure 8, top-right). Additionally, the GC from LC to VLPO did not change across LOR (Figure 8, bottom-left). Finally, during ROR, the GC from LC to VLPO significantly increased in post-ROR around 4–8 Hz (Figure 8, bottom-right, bottom-left, d = 0.67 ± 0.12, 95% CI = [0.47% ± 12.42% to 157.50% ± 59.94%]).

## Discussion

Although not fully understood, sleep and anesthesia represent two complex neural processes that alter consciousness (responsiveness). Parallel and systemic explorative analyses provide a unique opportunity to investigate the neuronal basis for both processes. Signals recorded in the present study from nuclei that are pivotal in sleep/wake modulation—VLPO and LC—during both WAKE and NREMS transitions and during isoflurane induced loss of responsiveness might enable us to identify converging or diverging patterns of underlying neuronal activities. Such findings also hold the potential to improve our general understanding about transitions across different brain states.

### Coherence during sleep and anesthesia

Coherence has been widely used to assess the functional connectivity or the integrity of neuronal connections between particular brain regions [42–44], quantifying the linear relationship between two signals based on similar frequency components [40, 45, 46]. Though not for specific nuclei, an increase in inter-hemispheric EEG coherence during NREMS has been shown decades ago [47] and it has been hypothesized that EEG coherence between brain areas is the manifestation of their functional connectivity. A concept that continues to be widely used today in neuroscience research [48, 49]. The current study showed that the coherence between the signals from VLPO and LC increased significantly during NREMS, when compared to WAKE. The increase in coherence during NREMS between VLPO and LC supports the direct physiological and functional connection between the two nuclei, as evidenced in previous reports [50, 51]. These studies have shown that VLPO acts as part of a network that facilitates sleep through GABAergic/galaninergic pathways, which have inhibitory control over nuclei that support wakefulness, such as the LC, and conversely, the LC inhibits the VLPO to promote wakefulness. In the current study, the increase and decrease in coherence between VLPO and LC, which are main players in the control of NREMS and WAKE, respectively, represent functional markers for these two states.

Coherence between VLPO and LC significantly decreased during post-LOR as compared to pre-LOR in the 3–8 Hz frequency range while in the WAKE → NREMS transitions, 3–5 Hz showed no change in coherence and 5–8 Hz showed an increase in the coherence. After LOR, VLPO and LC are less synchronous with each other in the dominant low frequency signal. The coherence between VLPO and LC across ROR did not change significantly. Unlike the alternating “flip-flop” pattern of coherence observed during the transitions from WAKE → NREMS and NREMS → WAKE [52] characterized by a complete reversal in the process of falling asleep and waking up, the emergence from anesthesia did not exhibit a reversal in the process of entering anesthesia. This has been previously established, showing that the emergence from GA is not a simple reversal of the induction process and may be potentially regulated by distinct neural circuits [53].

### ISPC during sleep and anesthesia

ISPC assesses the degree to which differences in oscillation phase angles between electrodes remain stable across multiple trials at each specific time and frequency point. Mathematically, ISPC is the length of the average vector of phase angle difference vectors from two electrodes [54]. When oscillations are in sync, their phase angle differences will remain constant [55]. During transitions from WAKE → NREMS and from NREMS → WAKE, the ISPC values followed the patterns of coherence values. The phase clustering between VLPO and LC was higher during NREMS and lower during WAKE. Higher ISPC between VLPO and LC during NREMS suggests a consistent alignment of their waveforms, pointing toward a more coordinated neuronal activity pattern, potentially contributing to the maintenance and stability of the state of sleep. Conversely, the decreased ISPC during WAKE may suggest a more dynamic and less stable neuronal interaction typical of an alert state [56].

During LOR and ROR, the ISPC values did not change across transitions. However, the ISPC values were very high at the slow-wave frequency range before and after LOR. Interestingly, particular forms of heightened interregional neuronal synchronization during anesthesia can disrupt the processing of meaningful information and lead to unconsciousness [57]. Studies have also shown that isoflurane-induced deep anesthesia enhances neuronal synchrony [58]. The results of the current study support these reports.

### GC during sleep and anesthesia

In general, GC is a commonly used statistical measure to assess directed functional connectivity between time series data, defined using predictability and temporal precedence [59]. Spectral GC quantifies the proportion of a signal's total power at a specific frequency that is influenced by another signal [60]. Bivariate GC may capture information from common sources and fail to differentiate direct and indirect information flow [61]. However, since sleep and anesthesia might involve long-range information flow through indirect pathways, including common sources could be beneficial in this context.

While the term “causality” may suggest that one variable directly causes another, it is crucial to understand that GC primarily offers insights into the predictability of variables. True causality between signals can only be established after accounting for the effects of other influencing signal sources within the framework [62]. Therefore, it is prudent to limit the interpretation to predictability or directional functional connectivity rather than inferring direct causation [62]. The present results showed bidirectional GC influence between VLPO and LC during WAKE and NREMS, re-affirming the reciprocal relationship between them. During NREMS, VLPO Granger caused LC in the slow wave (0.5–4 Hz) and the high (21–40 Hz) frequency range. Interestingly, during NREMS the GC from LC to VLPO reduced in the slow wave frequency range. This indicates a strong directional influence of VLPO on LC during NREMS, suggesting that LFP-signals originating from LC may be reliably predicted based on the preceding LFP-signals from VLPO. Additionally, during WAKE the GC from VLPO to LC reduced in the slow wave frequency range reaffirming the reciprocal inhibition.

During post-LOR, VLPO Granger caused LC at the slow-wave frequency range, like in NREMS. The VLPO is known to exert an indirect influence on thalamocortical circuits, which play a pivotal role in the generation of slow-wave oscillations [11]. By inhibiting arousal-related inputs, the VLPO facilitates the hyperpolarization of cortical and thalamic neurons, thereby establishing the conditions necessary for the synchronized neuronal firing that defines slow-wave oscillations [11]. This probably shows the engagement of VLPO in slow-wave oscillations rather than a direct involvement in the induction of anesthesia, as there is no tangible coherence or ISPC results. During post-ROR, LC Granger caused VLPO in the 4–8 Hz frequency range. As an effect of reduction of isoflurane concentration in the mouse brain, it is plausible that the arousal nuclei are activated after ROR. Activation of LC is known to enhance 4–8 Hz signal [63] which might influence VLPO.

### Unconsciousness versus unresponsiveness

In human as well as in animal research, transitions from WAKE to NREMS and *vice versa* can be clearly and precisely defined within the EEG. Additionally, there are established behavioral and electrophysiological methods to identify loss of consciousness driven by anesthetic agents in humans. However, in animal research, defining the exact point of loss (and recovery) of *consciousness*, is much less straight forward, even with the help of an EEG monitoring system. Defining such blurred transitions in the EEG within the time domain is part of the present research in our laboratory. Until we have a clear definition of the exact time point of any given transition, we suggest referring to these transitions as loss and recovery of “*responsiveness*” (LOR and ROR), instead of loss and recovery of “*consciousness,*” since the former is much easier to define in animal experiments. We are convinced that this approach provides a meaningful interpretation and discussion of the results.

## Conclusion

The functional connectivity dynamics between VLPO and LC exhibit a “flip-flop” pattern, indicating an alternating relationship during WAKE and NREMS. The transition from WAKE to NREMS and isoflurane-induced LOR showed diverging connectivity patterns between VLPO and LC. The coherence, phase synchrony and the GC influences between VLPO and LC showed divergent patterns for transition to NREMS and transition to LOR. This suggests that despite the behavioral and electrophysiological similarities, NREMS and isoflurane-induced LOR may involve at least partially distinct neuronal processes. The results additionally suggest that ROR and waking up from NREMS have distinct underlying mechanisms. This does not disregard the involvement of VLPO and LC in LOR and ROR. In our view, the extent to which the involvement of the nuclei in these transitions is passive or active during anesthesia, remains unclear.

## Limitations

Circadian rhythm exerting strong modulatory effects on sleep–wake dynamics [52] and anesthetic sensitivity, could in principle influence the interpretation of state-dependent neural connectivity. It can be speculated that WAKE-NREMS transitions occurring during the light versus dark phase might show differences in connectivity, reflecting circadian modulation rather than the transition itself. In the scope of the present study, however, VLPO—LC coherence was consistently observed to increase during NREMS across the entire recording period, indicating a robust state-dependent effect independent of circadian time. Transitions were therefore analyzed irrespective of circadian timing once this consistency had been established. Nevertheless, stratifying transitions by circadian phase or aligning baseline transitions and anesthetic testing within fixed circadian windows would be beneficial for advancing the understanding of how circadian regulation interacts with both sleep–wake transitions and anesthesia sensitivity.

Identifying LOR and ROR through behavioral testing by the experimenter introduced movement artifacts in the EEG and LFP recordings. This partially excluded critical data at these transitions from the analysis. Adopting alternative approaches, such as methods solely based on EEG analysis for identifying LOR and ROR, could facilitate sliding window analyses during these transitions, enriching the depth and precision of such studies. The precise moment of LOR (if the transition from responsiveness to unresponsiveness occurs in a similar time frame as of WAKE to NREMS transition) is still hard to determine through EEG markers, using conventional methods. It is plausible that sleep/wake-promoting nuclei could play a role in initiating the anesthetic state only at a certain, very narrow time point. However, identifying the exact transition and acquiring the corresponding data within the critical time frame may require advanced methodologies.

This study was designed understanding the transitions to and from unresponsive states resulting in the selection of data around LOR and ROR for analysis. While this method gave us a closer look into LOR and ROR, it introduced the limitations of fixed-dose isoflurane regimens without monitoring the dynamic relationship between anesthetic concentration and neural activity. Slight changes in isoflurane levels are known to significantly alter EEG patterns [64, 65] and failing to account for these fluctuations may obscure dose-dependent neural effects and limit the generalizability of the findings.

The results were interpreted on the assumption that the majority of the signals captured by LFPs are local electrical activity from the nuclei. LFPs are an important tool in neuroscience research but their interpretative accuracy is compromised by the phenomenon of volume conduction, which may include the integration of electrical signals from distant neural sources, thus confounding the identification of the precise spatial origin and challenging the assumption of locality in recorded activities [66, 67].

A critical factor lies in the cellular and functional heterogeneity of nuclei. It is well-established that only a subset of VLPO neurons actively promote NREMS, predominantly GABAergic and galaninergic cell populations, while neighboring regions and neuronal subtypes contribute variably to sleep–wake regulation [23, 68–73]. Glutamatergic neurons in the VLPO have been identified to promote wakefulness, destabilize NREMS, suppress REMS, and regulate cortical dynamics [22].

The majority of LC neurons are noradrenergic, which serve as the principal output cells and are classically described as wake-active [74, 75]. In addition, LC contains GABAergic neurons, which play an inhibitory role by suppressing the activity of neighboring noradrenergic neurons, acting as a brake on arousal to facilitate transitions into sleep states [76].

As mentioned earlier, LFPs, by their biophysical nature, reflect the aggregate synaptic and dendritic currents across a volumetrically broad region rather than isolated activity from sleep-promoting neurons alone [66, 77, 78]. Consequently, LFP signals recorded near the VLPO incorporate contributions from heterogeneous neuronal ensembles. This confounds direct attribution of observed LFP changes specifically to sleep-active VLPO neurons, highlighting the necessity for future investigations employing cell-type-specific techniques such as optogenetics, chemogenetics, or genetically encoded calcium indicators to disentangle these mixed signals [79, 80].

The present study exclusively used isoflurane as the anesthetic agent. Though the study gave insights about isoflurane induced unresponsiveness, given its specific pharmacodynamic properties, some observed effects may reflect drug-specific rather than state-general mechanisms. To identify if there are neural signatures that are agent invariant across unconscious states, future research should incorporate anesthetics with diverse molecular targets.

Original data are available from TF upon request.

## Supplementary Material

FINAL_Supplemantary_material_LJ1_TF4_zsaf287

## Data Availability

Raw data are available upon request to TF.
