# Supplementary material for: Non-rapid eye movement sleep and isoflurane-induced anesthesia show divergent subcortical connectivity patterns during transition phases in mice
Source: Sleep. 2025 Sep 24;49(4):zsaf287. doi: 10.1093/sleep/zsaf287 (PMC13089457; doi:10.1093/sleep/zsaf287)
Supplement: FINAL_Supplemantary_material_LJ1_TF4_zsaf287 [file final_supplemantary_material_lj1_tf4_zsaf287.docx]

**Non-rapid eye movement sleep and isoflurane-induced anesthesia show divergent subcortical connectivity patterns during transition phases in mice**

Leesa Joyce^1^, Rachel Nuttall^1^, Matthias Kreuzer^1^, Gerhard Rammes^1^, Gerhard Schneider^1^, Thomas Fenzl^1*^

1. Department of Anesthesiology & Intensive Care, School of Medicine and Health, Technical University of Munich, Germany

- Corresponding Author: Thomas Fenzl, thomas.fenzl@tum.de

**Author e-mail addresses**

leesamjoyce@gmail.com

rachel.nuttall@tum.de

m.kreuzer@tum.de

g.rammes@tum.de

g.schneider@tum.de

Supplementary material

At the end of the experiment, lesions were made through the LFP electrodes at VLPO, LC and VPM. As described in the methods section, the mouse brain was then perfused, sliced and Nissl stained to verify the LFP electrode targets. Black and white images of the Nissl-stained slices (right half) showcasing the corresponding regions in the mouse brain atlas ^31^ (left half) are shown below. The electrical lesions performed through the LFP are seen as dark stains spotted at the target locations. The lesion was done on the left hemisphere of the brain (The images are horizontally flipped during the slicing process). The images below are representative slices from one of the 12 mice.


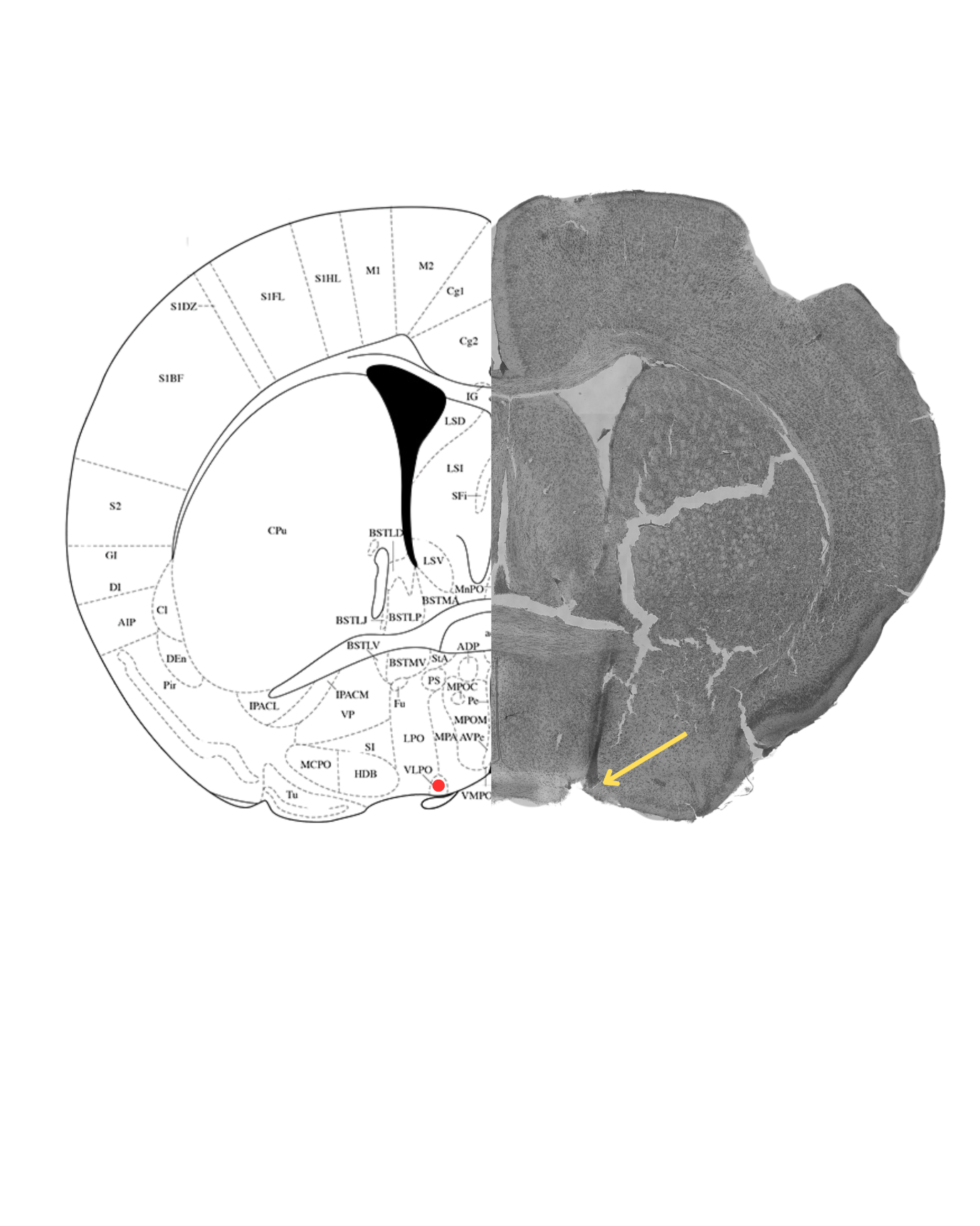


*Supplementary figure 1:* *The left half of the image shows a mouse atlas sketch of a coronal slice of the mouse brain, mapped to the corresponding regions in the brain where the target nuclei - VLPO is marked with a red dot. The right half of the image shows the corresponding coronal slice of a Nissl-stained mouse brain with a lesion at VLPO marked with a yellow arrow.*


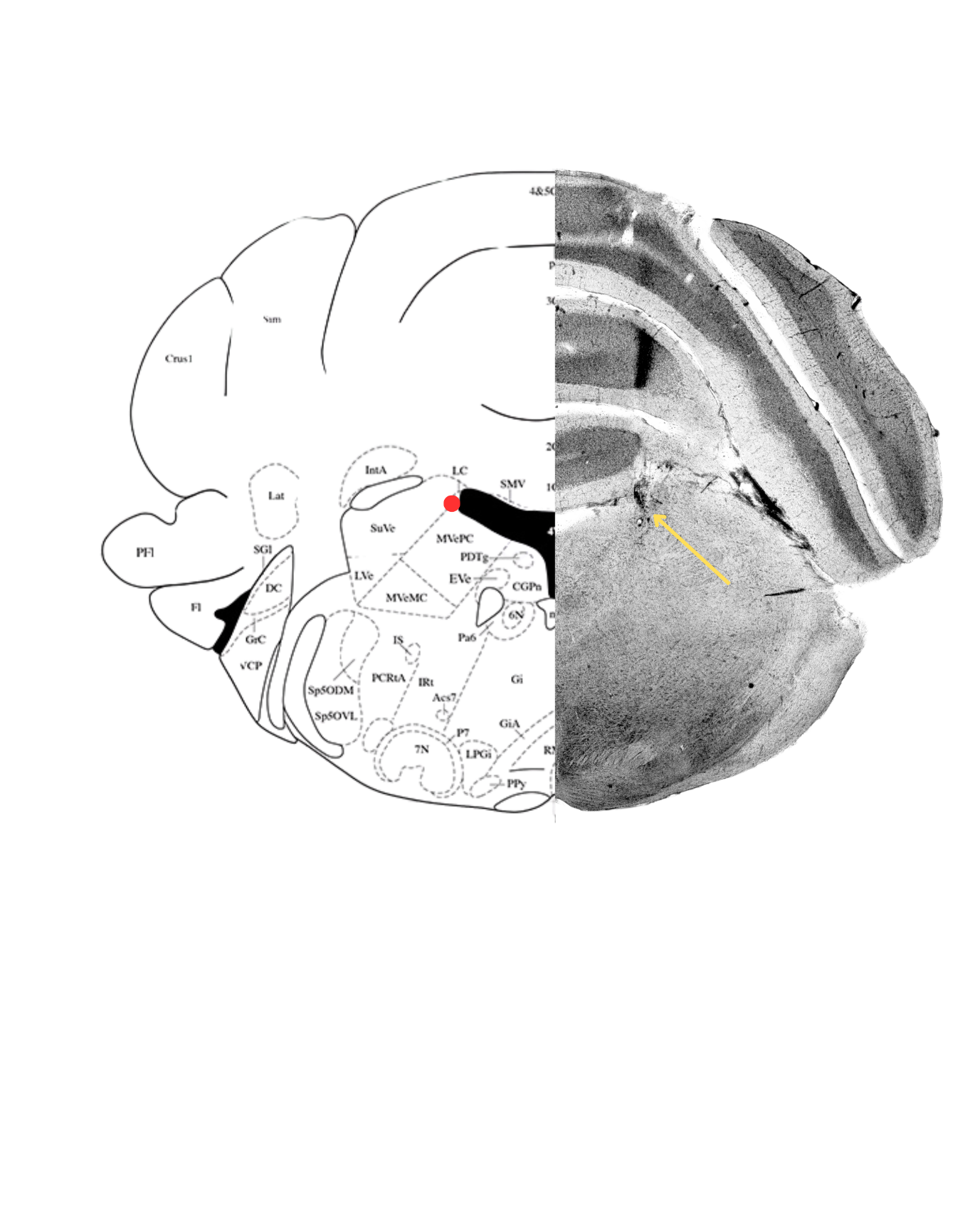


*Supplementary figure 2: The left half of the image shows a mouse atlas sketch of a coronal slice of the mouse brain, mapped to the corresponding regions in the brain where the target nuclei - LC is marked with a red dot. The right half of the image shows the corresponding coronal slice of a Nissl-stained mouse brain with a lesion at LC marked with a yellow arrow.*

*
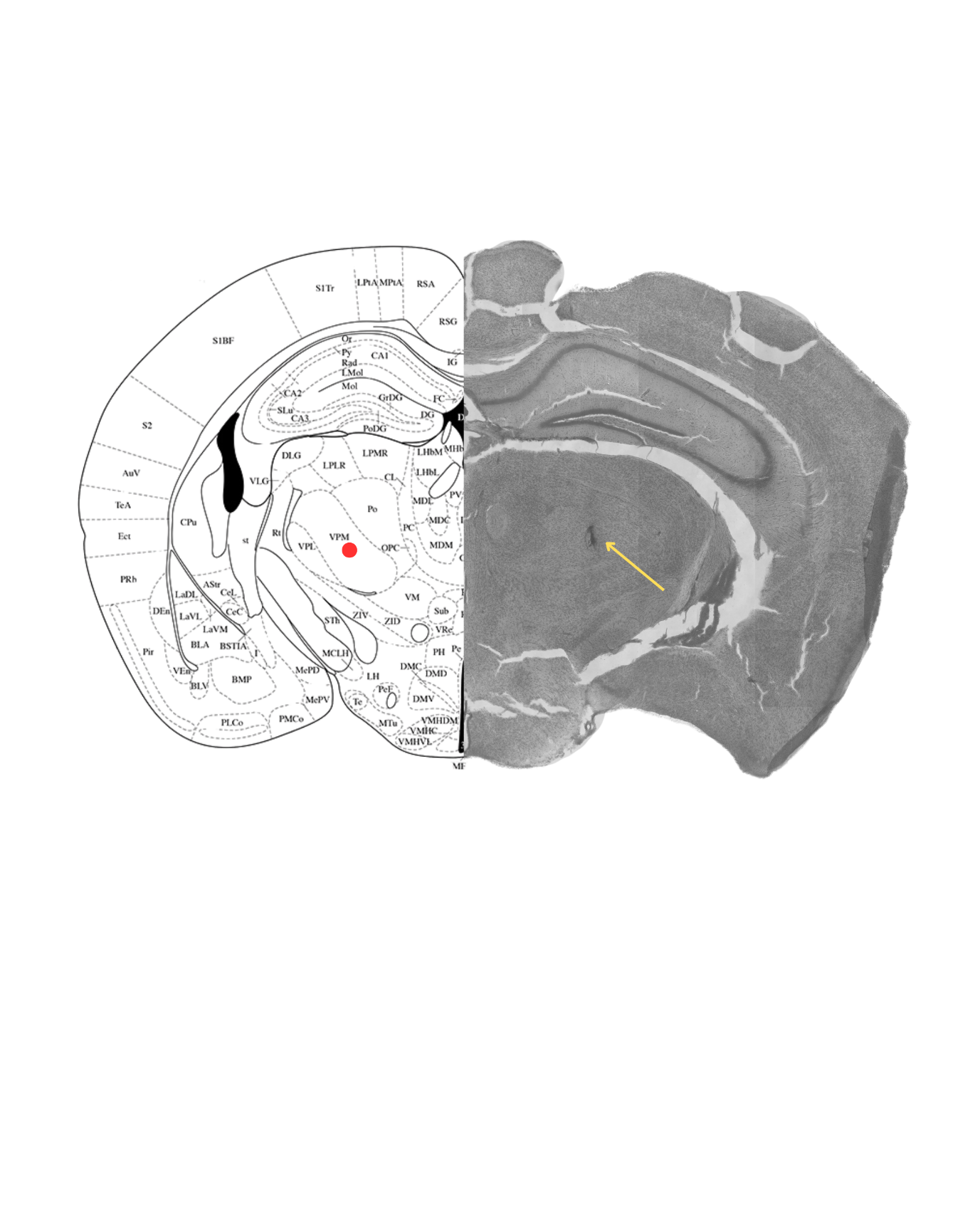
*

*Supplementary figure 3: The left half of the image shows a mouse atlas sketch of a coronal slice of the mouse brain, mapped to the corresponding regions in the brain where the target nuclei - VPM is marked with a red dot. The right half of the image shows the corresponding coronal slice of a Nissl-stained mouse brain with a lesion at VPM marked with a yellow arrow.*

Before analysing sleep transitions, 23 hours baseline recordings were sleep scored and the coherence between VLPO and LC was plotted against the hypnogram for all mice. For reference, coherence between VPM and LC was also plotted for corresponding time points. Both in active and inactive phases, the VLPO-LC pair showed consistent and prominent increase in coherence during NREMS.


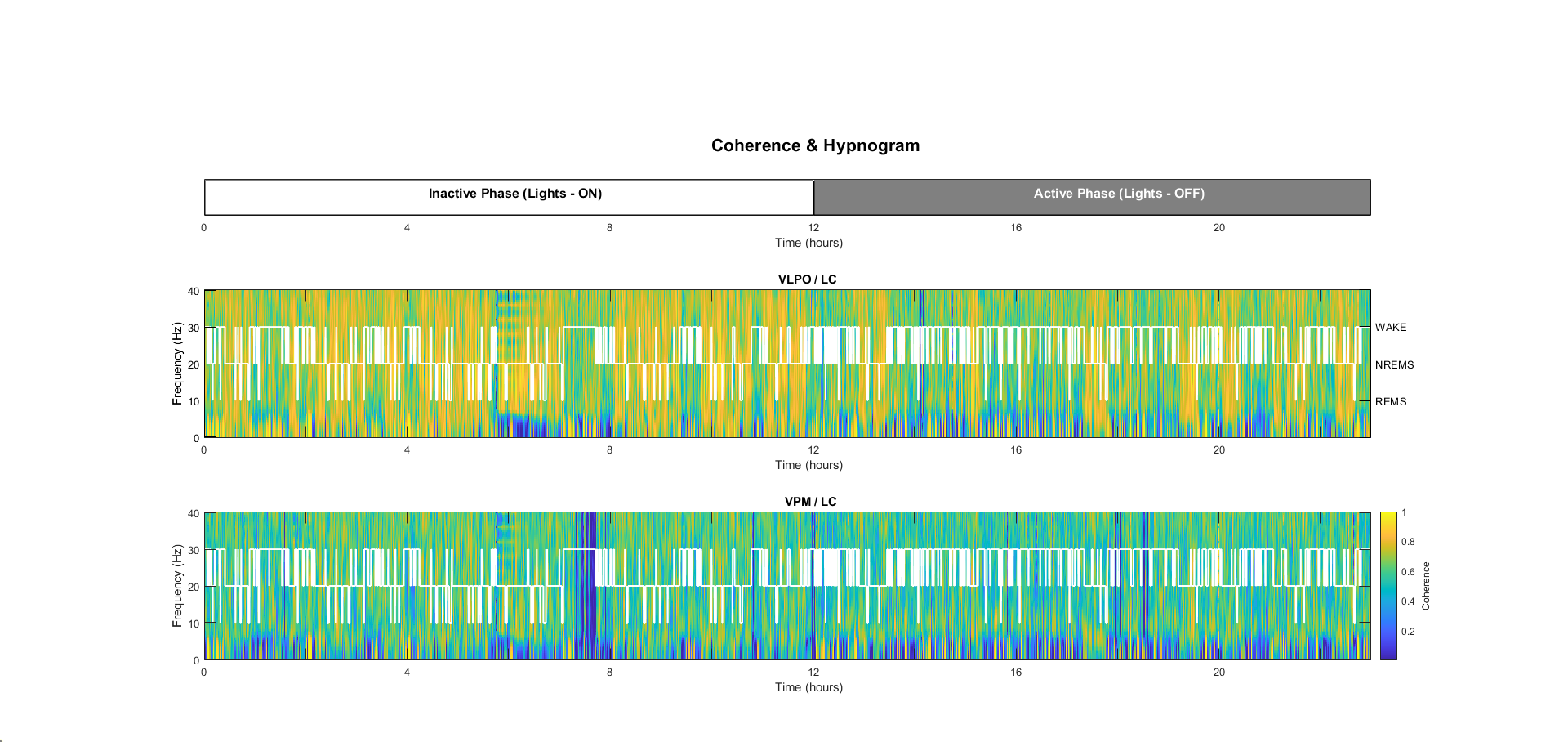


*Supplementary figure 4: A representative figure from a mouse showing coherence between VLPO-LC plotted with the corresponding hypnogram during 23 hours of baseline recording. VPM-LC coherence is also plotted along with the same hypnogram for reference. The colorplot shows coherence (yellow - high coherence, blue - low coherence) represented by the colorbar with the scale of coherence with varying colors. The y-axis shows frequency in Hertz and the x-axis shows time in hours. The colorplots are overlayed with white trendline hypnograms which are distinctly annotated as WAKE, NREMS and REMS on the right y-axis. The active (lights - OFF) and inactive (lights - ON) phases are denoted in grey and white colors respectively corresponding to the timeline on the x-axis. The figure shows that the VLPO-LC coherence increases during NREMS irrespective of the circadian cycle.*
